# Use of masks in public places in Poland during SARS-Cov-2 epidemic: a covert observational study

**DOI:** 10.1186/s12889-021-10418-3

**Published:** 2021-02-23

**Authors:** Maria Ganczak, Oskar Pasek, Łukasz Duda – Duma, Dawid Świstara, Marcin Korzeń

**Affiliations:** 1 Department of Infectious Diseases, Collegium Medicum, Uniwersytet Zielonogórski, Zielona Góra, Poland; 2 Collegium Medicum, Uniwersytet Zielonogórski, Zielona Góra, Poland; 3 Department of Methods of Artificial Intelligence & Applied Mathematics, Westpomeranian University of Technology, Szczecin, Poland

**Keywords:** SARS-Cov-2, Face mask, General population, Determinants

## Abstract

**Background:**

Face masks have been employed in the COVID-19 pandemic plans as a public and personal health control measure against the spread of SARS-CoV-2. In Poland, obligatory wearing of masks in public spaces was introduced on April 10th, 2020; a relaxation of previous universal measures was announced on May 29th, 2020, limiting use to indoor public places.

**Objective:**

To assess use of masks or other protective devices in public spaces in Poland during the SARS-Cov-2 epidemic.

**Methods:**

A non-participatory covert observational study was conducted on three dates, (10.05/18.05/25.05.2020) at public spaces in 13 regions with different risks. Ten consecutive individuals were observed by each of 82 medical students (*n* = 2460 observations), using a structured checklist.

**Results:**

Among 2353 observed persons, the female/male ratios were 1.0, 1.1, and 1.0 on the three dates. Almost three quarters - 73.6% (*n* = 552/750) were using masks on date 1, 66.5% (544/818) on date 2; and 65.7% (516/785) on date 3. Cloth masks predominated on all dates (64.7–62.3%-62.6%), followed by medical (23.4–28.5%-26.9%). Being female (OR = 1.77–1.47-1.53 respectively) and location in a closed space (OR = 2.60–2.59-2.32) were each associated with higher usage. Participants in sports were about two times less likely to use masks (OR = 0.64–0.53-0.53) as compared to other activities. The proportion using masks correctly decreased gradually over time (364/552; 65.9%; 339/544; 62.3% and 304/516; 58.9%). More females wore masks correctly (date 1: 205/294; 69.7% vs 159/258; 61.6%, and date 3: 186/284; 65.5% vs 118/232; 50.9%; *p* = 0.045; *p* = 0.0008 respectively). Uncovered noses (47.3–52.7%) and masks around the neck (39.2–42.6%) were the most frequent incorrect practices.

**Conclusions:**

Practices were not in line with official recommendations, especially among males, and deteriorated over time. Cloth masks were predominantly used in public spaces. Health promotion, through utilizing all available communication channels, would be helpful to increase compliance.

## Introduction

The severe acute respiratory syndrome coronavirus 2 (SARS-COV-2) pandemic that begun in the Chinese province of Hubei quickly became a global threat [1]. As of December 1st 2020, the virus has infected more than 62.8 million individuals worldwide and caused almost 1.5 million deaths [2]. With no effective pharmacological interventions or universal access to the vaccine, other modes of prevention are the best approaches to control the disease spread [3, 4].

Strategies to transition out of lockdown [5, 6], carried out in many countries, need to take into account both symptomatic and asymptomatic individuals in spreading SARS-CoV-2 from person-to-person through close contact [7, 8]. Protective equipment measures are needed [9, 10].

Either disposable surgical masks or reusable cotton ones are protective. Wearing masks in public places is seen as an important contribution to containing viral spread, especially when physical distancing is not possible or unpredictable [10]. Locations include stores and other shopping areas, workplaces, health care facilities (i.e. surgeries, hospitals, care-homes etc.), public transport, busy sidewalks, and households. If used widely and corresponding to risk, masks can reduce viral transmission. Benefits increase where exposure risk is high and are marginal where it is low [9, 10].

In the beginning of the pandemic, mask-wearing was controversial among leading health organizations, such as the World Health Organization (WHO) [11]. However, convinced by the evidence, the WHO has broadened its recommendations for use during the pandemic since June 5th, 2020 and advises that in areas where the virus is spreading, people should wear masks when social distancing is not possible [12, 13]. The US Centers for Disease Control and Prevention (CDC) has also modified previous recommendations, suggesting that, together with infected persons and health care workers, healthy people “should wear cloth face coverings in public settings when around people outside of their household” [14, 15].

The European Centre for Disease Control (ECDC) recommends use in the community, especially when visiting busy, closed spaces [16]. Formal guidance from the ECDC has informed official advice in many countries. As more was understood about asymptomatic transmission, governments were more inclined to mandate wearing and facilitate access. The latter was possible through various initiatives such as the provision of templates or instructions for citizens on how to make cloth masks at home, together with boosting domestic production or access to shops selling fabric and in supermarkets [17].

Different regulations have been in place in central Europe since the epidemic began, including Poland, Slovakia and Czechia [17, 18]. In many countries wearing became mandatory outside the home as part of physical distancing measures under lockdowns. In some other countries masks have been introduced as part of transition measures universally or in certain circumstances, such as on public transport or where physical distancing is not possible. In remaining countries, wearing is recommended, but not mandatory, as part of transitional measures [17].

In Poland, obligatory mask wearing was not introduced in the initial phase of the pandemic. On April 10^h^, 2020, 6 weeks after the country’s first laboratory confirmed SARS-Cov-2 case had been reported, a new control measure was announced [19]. It became obligatory to cover one’s nose and mouth in public spaces; any form of face covering was acceptable [18]. A relaxation of previous universal measures regarding wearing masks was announced on May 29th, 2020, limiting use to shopping areas, health care facilities, care-homes, public transport and churches [19]. The obligation to wear masks in indoor and outdoor public spaces has been restored since October 10th, 2020 [20].

Reports on use of masks by the general public as a preventive measure to limit transmission are scant, especially in European countries. Therefore, the objective of this study was to assess use of masks or other protective devices in public spaces in Poland during the epidemic and to evaluate influencing determinants.

## Methods

In Poland, mandatory face covering in public spaces was introduced in the beginning of April, 2020 [18]. In the end of May, 2020, it was limited to indoor public spaces [19]. A non-participatory covert observational study was carried out during three occasions over a period of three consecutive weeks (10.05.2020, 18.05.2020, 25.05.2020) among the general public in Poland from the different risk areas regarding SARS-Cov-2 pandemic. The risk classification was adapted from a report made by the Ministry of Health on basic reproductive number (R) estimates [21]: high-risk areas (R > 1); low-risk areas (R 0.5–1.0); very low-risk areas (R < 0.5).

According to the latest census, Poland has a population of 38,383,000 [22]. The representative target sample size needed to achieve the study objectives and sufficient statistical power was calculated with a sample size calculator [23]. This arrived at 664 participants, using a margin of error of ±5%, a confidence level (CI) of 99% and a 50% response distribution.

At 2 pm, at each time point described above, eighty two 4th year medical students observed ten consecutive individuals of > 4 years of age while venturing out into public spaces (820 observations per time point) which were divided into two categories: open/enclosed space. The age frame > 4 years was arbitrarily chosen due to the governmental regulation requiring mask use for children older than 4 years and adults [18]. Two types of public spaces were chosen arbitrarily: 1. representing open space, i.e. “sidewalks/parks” and 2. representing enclosed space - “shopping centers”. Three activities were observed: walking, outdoor sport activities and shopping. Students were randomly selected to make observations outdoors (75% observations) and indoors (25% observations). All 4th year medical students from the University of Zielona Gora, Poland, participated in the study collecting the relevant data. Trained students used a validated structured checklist, as recommended by “WHO guidelines on the use of masks in the context of COVID-19” [24]. Rational use of a mask was defined as wearing a specific type of mask (N95 respirator, medical mask, cloth mask, face shield or other – a scarf/bidon). The correct manner of masks use in the public was defined as the use of masks as per the WHO/ECDC guidelines [16, 24]. Observations were carried out in student residence locations without the knowledge of those observed (covert).

### Statistical analysis

Field notes were taken at observation points. Next, data were entered to MS Excel 2013. Data quality was assured by the review of data completeness by the research team (OP and MG). Data were validated using STATISTICA PL Version 12.5 (StatSoft Inc., 2016). Our main outcome was mask usage. For socio-demographic variables, gender was coded as one for males, and two for females. A place of residence variable was arbitrarily divided into 2 categories: a city up to 50,000 citizens and > 50,000 citizens; a region regarding pandemic risk – into: high-risk (R > 1) and low-risk/very low-risk areas (R ≤ 1.0); a type of activity observed – into playing sports/other activities. A bivariate analysis assessed the demographic characteristics (gender, residency and region regarding pandemic risk), together with the participant location during the observation (open/closed space), and the type of activity observed, associated with the outcome variable. The categorical variable groups were compared using the chi-square test with Yates correction and Fisher’s exact test. For observations made at each of the 3 time points, standard single-outcome logistic regression models were built for the predicted outcome variable (mask usage); all models were then reduced by the use of a stepwise selection and a backward procedure [25] with the help of R software [26]. Unstandardized regression coefficients in the regression model were used to assess any change in the model. A change in coefficients was compared and used to determine any variable change. For binary data the exponent of the coefficient is interpreted as the odds ratio (OR) [27].

## Results

### General characteristics of study participants by time point

The general characteristics of study participants by each time point is presented in Table 1. Seven medical students were on a sick leave at the first time point and three at the third, which limited the observations number to 750 on May 11th and to 785 on May 25th. As some observations were excluded due to incomplete data, the total number of observations was 2353 (range: 750–818). The female/male ratio was similar (1.02; 1.13 and 1.03) at the 3 time points; this was similar to that observed in the general population (1.07) [22]. At each time point, the number of observations made in the open space was similar (78.1, 73.5 and 74.5%); “walking” was the most common activity (51.5, 47.4 and 50.7%), followed by “shopping” and “playing sports”.

**Table 1:** Respondent characteristics and mask usage by selected variables at three time points. Poland 2020; *n* = 750–818

Variable		11.05					18.05					25.05				
		Total		Mask usage			Total		Mask usage			Total		Mask usage		
		N^a^ 750	%	n	%	p	N^a^ 818	%	n	%	p	N^a^ 785	%	n	%	p
Gender	Female	371	49.5%	294	79.2%	0.0005	434	53.1%	318	73.3%	< 0.0001	398	50.7%	284	71.4%	0.0008
	Male	379	50.5%	258	68.1%		384	46.9%	226	58.9%		387	49.3%	232	59.9%	
Residence	Town ≤50,000 inhabitants	395	52.7%	302	40.3%	0.06	445	54.4%	296	36.2%	0.99	387	49.3%	264	33.6%	0.15
	Town > 50,000 inhabitants	355	47.3%	250	33.3%		373	45.6%	248	30.3%		398	50.7%	252	32.1%	
Region	R^b^ < 0.5	220	29.3%	160	72.7%	> 0.65	269	32.9%	164	61.0%	> 0.004	277	35.3%	171	61.7%	> 0.055
	R 0.5–1.0	410	54.7%	302	73.7%		428	52.3%	306	71.5%		389	49.6%	268	68.9%	
	R > 1.0	120	16.0%	90	75.0%		121	14.8%	74	61.2%		119	15.2%	77	64.7%	
Observation	Open space	578	78.1%	407	70.4%	< 0.0001	585	73.5%	359	61.4%	< 0.0001	577	74.5%	351	60.8%	< 0.0001
	Closed space	162	21.9%	141	87.0%		211	26.5%	179	84.8%		198	25.5%	159	80.3%	
Activity^a^	Walking	353	51.5%	261	73.9%	< 0.0001	351	47.4%	235	67.0%	< 0.0001	368	50.7%	243	66.0%	< 0.0001
	Playing sports	154	22.4%	97	63.0%		170	23.0%	91	53.5%		150	20.7%	73	48.7%	
	Shopping	179	26.1%	152	84.9%		219	29.6%	179	81.7%		208	28.7%	167	80.3%	

^a^the number is smaller than the number of the total observations due to the difficulties in classifications

^b^basic reproductive number

### Mask usage determinants by time point

**Fig. 1:**
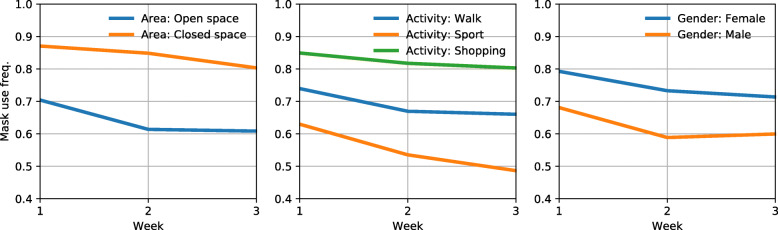
Masks usage trend by area, activity and gender. Poland, 2020; *n* = 750–818

The percentage of those using masks was 73.6% (552/750) at the first time point, decreasing a week later (66.5%; 544/818) and then – 2 weeks later (65.7%; 516/785). Table 1 and Fig. 1 present masks usage by the public at the three time points by selected variables.

Significantly more females than males used masks (*p* < 0.01 for the three time points). A decreasing trend was observed in relation to mask use at three time points for both: the general public and females. In the case of males, a decrease in usage was seen during the second observation (68.1% vs 58.9%), however, the percentage of those using masks increased slightly (59.9%) a week later.

Masks usage in both - enclosed and open space decreased among study participants during the 3-week observation (87.0, 84.8, 80.3, and 70.4%, 61.4, 60.8% at the 3 consecutive time points). A statistically significant difference (*p* < 0.01) was observed between masks use in the open/enclosed locations at the three time points; Table 1, Fig. 1.

The percentage of people wearing masks at three time points in relation to the activity performed is presented at Table 1 and Fig. 1. The compliance with mask use was the highest while shopping (84.9%; 81.7 and 80.3%) and the lowest during outdoor sport activity (63%; 53.5 and 48.7%). A statistically significant difference was observed in masks usage between activities at the three time points (*p* ≤ 0.01). The decrease of mask usage over time was more evident among those in sports than among the other activities.

As reported in Table 1, at the 2nd time point those living in low risk areas (R 0.5–1.0) were more compliant regarding masks usage compared to regions with higher (R > 1.0) and very low (R < 0.5) pandemic risk (*p* = 0.004). There were no statistically significant differences observed in masks usage at any of three time points (*p* > 0.05) regarding the place of residence.

Multiple logistic regression analysis showed that participant gender, location during the observation and type of activity were independent predictors for mask usage (Table 2). Females were more likely to wear masks (for 3 time points: OR = 1.77; OR = 1.47; OR = 1.53, *p* = 0.002; *p* = 0.03; *p* = 0.01 respectively). Participants observed in the closed spaces were more likely to use masks than those outdoors (OR = 2.60; OR = 2.59; OR = 2.32*, p* = 0.0006; *p* < 0.0001; *p <* 0.0001 respectively). Interestingly, participants in sports were about two times less likely to use masks as compared to other activities. (OR = 0.64; OR = 0.54; OR = 0.54, *p =* 0.03; *p* = 0.0007; *p* = 0.001 respectively).

**Table 2:** Logistic regression model: association of the mask usage with selected variables by time point (odds ratio (OR) estimates and 95% confidence intervals (CIs) of OR estimates); Poland, 2020; *n* = 750–818

Variable	Time point 1		Time point 2	Time point 3		
	OR^a^	CI	OR	CI	OR	CI
Gender: female	1.77	1.24–2.53	1.47	1.05–2.00	1.53	1.07–2.12
Area: closed space	2.60	1.54–4.58	2.59	1.69–4.06	2.32	1.54–3.59
Activity: sport	0.64	0.43–0.97	0.54	0.36–0.76	0.54	0.36–0.78

^a^Odds ratio (OR) = ratio between the two categories tested in each variable, controlling for other variable

### Correct mask usage over time

The proportion using masks correctly decreased gradually over time (65.9%; 364/552, 62.3%; 339/544 and 58.9%; 304/516 respectively). A statistically significant between-gender difference was noted in the 1st and 3rd week of observation, with more females wearing masks correctly (1st week: 205/294; 69.7% vs 159/258; 61.6%, 2nd week: 197/318; 61.9% vs 142/226; 62.8% and 3rd week: 186/284; 65.5% vs 118/232; 50.9%; *p* = 0.045; *p* = 0.83; *p* = 0.0008 respectively). The differences in the correct manner of masks use were not significant regarding location and activity (*p* > 0.05).

### Type of mask used

A predominance of cloth masks was observed at all 3 time points followed by medical masks (Table 3); face shields were the type of face protection used the rarest.

**Table 3:** Frequency of mask usage by type of mask used and time point; Poland, 2020; *n* = 750–818

Type of mask	11.05				18.05				25.05			
	Usage		Correct usage		Usage		Correct usage		Usage		Correct usage	
	n	%	n	%	n	%	n	%	n	%	n	%
Cloth mask	357	64.7%	231	64.7%	339	62.3%	204	60.2%	323	62.6%	176	54.5%
Medical mask	129	23.4%	83	64.3%	155	28.5%	102	65.8%	139	26.9%	84	60.4%
N95 respirator	20	3.6%	16	80.0%	12	2.2%	10	83.3%	18	3.5%	17	94.4%
Face shield	12	2.2%	11	91.7%	7	1.3%	6	85.7%	8	1.6%	8	100.0%
Other^a^	34	6.2%	23	67.6%	31	5.7%	17	54.8%	28	5.4%	19	67.9%

^a^scarf/bidon

The correct manner of mask use by type and time point is presented in Table 3. During the 1st week, 80.0% were correctly wearing N95 respirator mask with an increased correct use in the 2nd and 3rd week (83.3 and 94.4%). An opposite trend had been observed for medical and home-made masks: 64.3%; 65.8%; 60.4 and 64.7%; 60.2%; 54.5% respectively were using those masks at the 1st, 2nd and 3rd week. There was not statistically significant between-type difference found at the 1st and 2nd time point regarding the correct wearing of a given type of a mask (*p* > 0.11); however, more participants used N95 respirators correctly when compared to other types of masks (*p* = 0.0008) at the 3rd time point. At all 3 time points uncovered noses (89/188; 47.3%, 108/205; 52.7% and 106/212; 50%) and masks around the neck (80/188; 42.6%, 85/205; 41.5% and 83/212; 39.2%) were the most common incorrect practices, followed by touching masks by hands (9/188; 4.8%, 8/205; 3.9% and 14/212; 6.6%) and incorrect mask fixation (8/188; 4.3%, 4/205; 2% and 9/212; 4.2%).

## Discussion

### Results overview

Overall, compliance with wearing masks by the general public in Poland was good in the beginning of the study period (74%), however, it decreased (to 66%) in the 2nd and 3rd week of the observation, with a predominance of cloth masks observed. Female gender and closed space were each associated with higher mask usage. Those playing sports had about two times lower chance to use masks. The percentage of those using masks in the correct manner also decreased gradually over time, mainly due to a decreasing trend observed for medical and cloth masks; significantly more females correctly used masks. Breaches in nose covering and hanging masks around the neck were the most common incorrect practices while wearing a mask.

### Trends in mask use

The willingness of the general public plays a decisive role in achieving the successful implementation of protective measures [3]. However, it is still a problem to encourage the public to unconditionally and continuously follow these recommended preventive actions.

The study found that in May 2020, when wearing masks in public spaces was compulsory in Poland, face covering was relatively high (74%). According to Ipsos [28], in the initial phase of the pandemic (a week after the first case had been reported in Poland), only 4% of randomly selected Poles reported wearing masks in public settings to protect from coronavirus. Two weeks later, the percentage had increased 5-fold. Five weeks later, 2 weeks after it became obligatory to cover one’s nose and mouth in public in Poland, 78% of respondents stated they used masks “always” or “sometimes”.

Concerns about the pandemic, combined with the perceived risk of infection may explain the high rates of masks use in our study. The strong association between respondents’ risk perceptions and taking comprehensive precautionary measures against influenza, Middle East Respiratory Syndrome (MERS) and Severe Acute Respiratory Syndrome (SARS) infections has been reported previously [29–32]. Apprehensions about relatively high administrative penalties while not complying with governmental regulation on wearing masks could also play a role in the high percentage of those observed using masks at the beginning of this study.

Other studies found extreme variation in COVID-19-related face coverings worldwide [33, 34]. As the example, in the report of the prevalence of face coverings in public places Elachola et al. found that it was almost universal in Cambodia (97%) and Peru (86%), however, much lower in India (41%), Mexico (25%) and USA (21%); Democratic Republic of Congo showed the lowest rate (4%) [33]. In the same vein, in a study that evaluated the uptake of non-pharmaceutical measures during the pandemic influenza A (H1N1) of 2009, participants of Asian origin had the higher facemask uptake (71%) compared to the uptake of participants of Western or Latin American origin [35]. In many Asian countries, the SARS epidemic in 2002, the pandemic influenza A (H1N1) of 2009, along with overcrowding, dense smog and air pollution may explain the high uptake of facemasks; the transition to wearing face covering during the COVID-19 pandemic was then acceptable and easier [33, 36]. The differences in face covering prevalence may be also attributable to variation in existence of recommendations, availability and affordability of masks, as well as delayed endorsement of masks as a COVID-19 mitigation tool [33].

The concept of collective responsibility versus personal freedom has been important in the context of face masks usage since the beginning of this pandemic. According to Gostin [37], compulsory public health prerogatives should be assessed and justified under a common ethical standard. This means individuals must pose a significant risk of spreading a dangerous infectious disease, such as SARS-Cov-2, interventions, e.g. mask use, must be likely to minimize risks, use of compulsion should be proportionate to the risk, and assessments must be based on the best available scientific evidence. In this context, in the interim guidance released on December 1st, 2020, WHO recommends that decision makers should apply a risk-based approach when considering the use of masks for the general public [13]. The relatively high face covering prevalence observed in this study may suggest that collective responsibility can take precedence over the personal freedom when the belief of potential infringement of civil liberties resulting from compulsion is counterbalanced by the potential health benefits on the population level.

The decreasing trend regarding mask usage by the general public in Poland through the 3 weeks of observation, which illustrates behavioral change, may be due to a gradual decrease in concerns about the pandemic over time and lowering in the perceived SARS-Cov-2 infection risk. Less strict supervision, monitoring and inadequate governmental regulation enforcement by the relevant services may also influence the rapid decrease in compliance rates. Although failure to comply with the government regulation could result in a fine of up to Polish zloty (PLN) 500 to PLN 30,000, according to the National Police Headquarters, between April 15th and May 31st 2020, 40,000 citizens were instructed on correct mask use; only around 13,000 were fined for not using a mask in the public space, around 5000 applications were made to the court to fine individuals for not wearing a mask [38].

Numerous examples of political leaders, including the Polish president and the prime minister not using masks in public spaces, which was transparently reported by the media [39–41], could influence mask usage by the public, by simply signaling that fears related to the virus are overblown. Public officials should be acutely aware they are in position to lead by example, with the choices they make and how they carry themselves can send a powerful message; this also refers to the SARS-Cov-2115 pandemic. Sadly, masks have become one of the potent symbols in how poorly Polish leaders have responded to this public health crisis.

### Determinants of mask use

Various factors, including socio-demographic characteristics, social context and individual values can affect the subjects’ perception of their actual risk of disease, as well as influence their worries about a pandemic [3, 42, 43]. Based on recent studies, being female is associated with a higher chance of adopting protective behaviors [3, 30, 44]. The results of this study show that males, traditionally risk takers, were significantly less likely to have appropriate practices regarding mask usage, and protecting themselves and others against SARS-Cov-2. These findings are consistent with other studies on SARS and MERS, showing that females are more health conscious and risk averse [30–32]. This was also reported during the current SARS-Cov-2 pandemic [45]. It is suggested that health promotion messages sent through female mediators who are significant for young males, e.g. mothers, sisters or partners, could increase mask use for this vulnerable subgroup [31].

Although not assessed in this study, younger respondents are reported as less likely to use masks in order to mitigate the chances of spreading and contracting SARS-COV-2 [46]; this might contribute to the high incidence of confirmed SARS-Cov-2 infections among that group [47]. This poses a danger not only to their peers but also to vulnerable groups, such as the elderly, those with comorbidities, people in long-term care.

Wearing masks when out in public was significantly lower in open spaces during the three-week observation, and decreased to a greater extent (10%) over time, when compared to enclosed spaces. During the study period government officials announced that in the upcoming weeks it would not be necessary to wear masks while walking on the street, in parks or during sport activities outdoors, e.g. riding a bike [48]; this could have influenced the behavioral change regarding the mask usage by the general public. The message that it would still be mandatory to wear masks in shops might have made the population consider its importance, which in turn influenced high compliance.

### Type of mask used

To control the infection source, as well as to self-protect, cloth masks, as recommended by the WHO, CDC, and ECDC [13, 14, 16] are likely to be adequate to minimize SARS-Cov-2 transmission in the community, especially if everyone wears a mask correctly. Cloth masks were predominantly used in the public space in this study, possibly due to the low price and the fact that they can be easily manufactured or made at home and reused after washing. The shortage of mask supply in the community and the integrated governmental and media message that medical masks and N95 respirators must be reserved for health-care workers might have influenced their poor usage by the public.

Notably, compliance of correct mask use was poor – only two thirds of those wearing masks (cloth and medical) wore them correctly at the first time point, this decreased over time, with 55% wearing cloth masks and 60% - medical ones. Although N95 respiratory masks were rarely used by the public, compliance with correct use was the best and increased over time. One of the possible interpretations of this observation could be that the potential N95 respiratory masks wearers were more health oriented and health educated. People with high health literacy skills are more competent in related to public health outbreak controls [3, 44, 49]. Due to the small number of observations regarding this type of mask usage, further qualitative and quantitative studies are needed to better assess this issue. Correct mask usage was gender dependent with more females using a mask in the proper manner. Interestingly, no differences were observed regarding the correct mask usage and residence, as well as location and type of activity.

At all 3 time points breaches in nose covering and hanging masks around the neck were the most common incorrect practices while wearing a mask. Such practices elevate infection risk regarding SARS-Cov-2 and other airborne respiratory pathogens. Notably, the pathogens may settle on the surface of used masks layers, resulting in mask contamination [50] which in turn highlights the risk of self-contamination to the wearer, particularly when fixing, elevating or doffing a mask.

### Strengths and limitations

To our knowledge, this is the first study to investigate masks use in the general population in Poland, as well as in the European Union (EU). The mask usage has been assessed by a non-participatory covert observational study. Other methods to assess the uptake of facemask against respiratory infections, include observational studies (most of them standard interviewer-administered surveys) and cases series (focus groups or qualitative surveys) [36]. Nevertheless, such quantitative and qualitative studies are based on reliability of reported preventive measures taken by interviews; this may be questionable [51]. Photo-epidemiology, which involves quantification of facemasks through photo frames from surveillance camera, is a valuable method of assessing the prevalence of mask use at the population level [33, 34, 52]. With the advent of hand-held devices it can help to assess face mask use in crowds where other methods of assessment are challenging, e.g. at mass gatherings [52], at the airports [34], and at the venues such as groceries, markets and commodity food distribution centers [33]. One of the disadvantages of this method is its inadequacy to verify the type of facemask used, such as loosely fit surgical masks or fit-tested N95 masks [34].

One particular strength of this study is that participants were observed during an actual continuing outbreak - data collection started 3 weeks after an introduction of the regulation about obligatory mask use in the public space. Furthermore, by covert observation, the Hawthorne effect has been limited as the behavior of participants was not altered [53]. In addition, the method used helped to verify the type of mask used, as well as compliance of correct mask use. However, the study has some limitations which should be pointed out. Community-based national surveys were not feasible regarding the phase of SARS-Cov-2 pandemic in Poland in which the study had been conducted due to the reallocation of funds and resources to the pandemic-oriented issues. As such, data were collected through students observations, depending on their place of residence. Therefore, the majority of the participants were observed in the western and central regions of Poland, where most of the students came from. Further research should cover all Polish provinces to better assess mask usage at a national level. Due to student sick leave, the numbers of observations were smaller at the first and third time point than at the second time point. Nevertheless, the response rate was still high which led to a relatively large sample size. Another limitation is that the study was administered over a short time period. Thus, the stability of the responses is unknown [31]. Further observations are needed to track possible changes as the pandemic evolves. The implication is for future research to assess whether there is a relationship between mask usage and other variables, not covered by this study, such as education, knowledge on infection control, anxiety level, trust in the health authorities, etc. Finally, this study did not address causation. Therefore, the regression results should be interpreted with caution.

## Conclusions

The results show an essential difference in how the general public responds to the SARS-Cov-2 pandemic in the context of mask usage. Practices were found to be inadequate, especially among males, and tended to decrease over time, more significantly among those wearing cloth masks than when compared to those wearing N95 respirators. This message can be used to target specific vulnerable groups when developing public health campaigns which should be then rigorously evaluated for their effectiveness. Among those using masks, every third individual wore them incorrectly. Therefore, awareness campaigns regarding the need for the proper usage of face masks - by utilizing all communication channels available - would be helpful during this pandemic [46]. The results may also help policy makers to adequately tailor mask usage strategies to better prevent SARS-Cov-2 transmission. Finally, political leaders should act as role models and set a good example wearing masks in the public arena to create a sense of solidarity and make a gesture with regards to evidence based practices.

In conclusion, this study identified topics that may need attitudinal modification to improve self-protection against SARS-Cov-2 infection and community spread of the virus. There is now an ideal moment to implement these adjustments.

## Data Availability

The datasets used and/or analyzed during the current study are available from the corresponding author on reasonable request.

## Abbreviations

CDC: Centers for Disease Control and Prevention
CI: Confidence interval
COVID-19: Coronavirus Disease 2019
ECDC: European Centre for Disease Prevention and Control
EU: European Union
MERS: Middle East Respiratory Syndrome
OR: Odds ratio
PLN: Polish zloty
R: Basic reproductive number
SARS: Severe Acute Respiratory Syndrome
SARS-Cov-2: Severe Acute Respiratory Syndrome Coronavirus 2
WHO: World Health Organization
